# Complete Chloroplast Genome Sequence of* Justicia flava*: Genome Comparative Analysis and Phylogenetic Relationships among Acanthaceae

**DOI:** 10.1155/2019/4370258

**Published:** 2019-08-06

**Authors:** Samaila S. Yaradua, Dhafer A. Alzahrani, Enas J. Albokhary, Abidina Abba, Abubakar Bello

**Affiliations:** ^1^Department of Biology, King Abdulaziz University, Jeddah, Saudi Arabia; ^2^Centre for Biodiversity and Conservation, Department of Biology, Umaru Musa Yaradua University, Katsina, Nigeria

## Abstract

The complete chloroplast genome of* J*.* flava*, an endangered medicinal plant in Saudi Arabia, was sequenced and compared with cp genome of three Acanthaceae species to characterize the cp genome, identify SSRs, and also detect variation among the cp genomes of the sampled Acanthaceae. NOVOPlasty was used to assemble the complete chloroplast genome from the whole genome data. The cp genome of* J*.* flava *was 150, 888bp in length with GC content of 38.2%, and has a quadripartite structure; the genome harbors one pair of inverted repeat (IRa and IRb 25, 500bp each) separated by large single copy (LSC, 82, 995 bp) and small single copy (SSC, 16, 893 bp). There are 132 genes in the genome, which includes 80 protein coding genes, 30 tRNA, and 4 rRNA; 113 are unique while the remaining 19 are duplicated in IR regions. The repeat analysis indicates that the genome contained all types of repeats with palindromic occurring more frequently; the analysis also identified total number of 98 simple sequence repeats (SSR) of which majority are mononucleotides A/T and are found in the intergenic spacer. The comparative analysis with other cp genomes sampled indicated that the inverted repeat regions are conserved than the single copy regions and the noncoding regions show high rate of variation than the coding region. All the genomes have* ndhF* and* ycf1* genes in the border junction of IRb and SSC. Sequence divergence analysis of the protein coding genes showed that seven genes (*petB, atpF, psaI, rpl32, rpl16, ycf1, and clpP*) are under positive selection. The phylogenetic analysis revealed that Justiceae is sister to Ruellieae. This study reported the first cp genome of the largest genus in Acanthaceae and provided resources for studying genetic diversity of* J*.* flava *as well as resolving phylogenetic relationships within the core Acanthaceae.

## 1. Introduction

*Justicia* L. is one of the largest and the most taxonomically complex genus in Acanthaceae belonging to the tribe Justicieae consisting of ca. 600 species [1–3] typified by* J. hyssopifolia* (Sp Pl. 15 1753; Gen. Pl. ed. 5, 10 1754; nonsensu Nees 1832, 1847; nec sunsu Kuntze, 1891) distributed in the tropical and subtropical regions of the world, extending into warm temperate zones in Europe, Asia, and North America [4]. The genus is the type specimen of the tribe Justiceae and is characterized by a number of characters which includes corolla having 2 libbeds (bilobed upper lip and trilobed lower lip) and 2 bithecous stamens, one of the two named thecae above the other and the other one contains stur at the base [1, 3]. Several authors [5–7] include small segregate genera in the genus while author [1] in his own view reported a new classification adding more taxa and defined the genus as having 16 sections based on some floral parts and seeds. Recent molecular studies reported that* Justicia* s. l (Graham 1988) is paraphyletic [8, 9]. Lindau classified* Justicia* and several closely related species under a tribe that is characterized by androecium having 2 stamens and Knotchenpollen. There is a problem with this system of classification as reported by some student of Acanthaceae because some species in the tribe do not have this character while several taxa with the characters were classified in other tribes. However, there is still a problem in the status of the tribe Justiceae; [6] divided Ruellioideae into Justicieae, Ruellieae, and Lepidagathideae, whereas authors [10] in their study on phylogenetic relationship among Acanthaceae using* trnL-trnF* reported* Ruellia* and* Justicia* as separate lineages. Recently, [11] classified the member of the Justicieae as subtribe under the tribe Ruelloideae.

*Justicia flava* is among the endangered species in Saudi Arabia; the plant is widely used in traditional medicine in treating various ailments such as cough, paralysis, fever epilepsy, convulsion, and spasm and skin infection disorder. The roots are also reported to be used in treating diarrhea and dysentery [12, 13]. As reported by [14] the plant has antimicrobial and antioxidant activity as well as wound healing activity. Despite the endangered nature and uses in traditional medicine of the species, the complete chloroplast genome of the species was not sequenced until this study.

Comparison of complete chloroplast genome provides very informative information for reconstruction of phylogeny and resolving evolutionary relationships issues at various taxonomic levels [15–19]. This is as a result of the conservative nature of the chloroplast genome [20]; this conservative nature is because the plastome evolves about half the rate of other genomes like the nuclear [21, 22]. However, rearrangements in the sequence of chloroplast genome were reported by various plant chloroplast genome studies [19, 23–25]. These rearrangements occur as a result of contractions, expansions, and inversions in the single copy regions (large single copy and small single copy) and the inverted repeats [26, 27]. The rearrangements of the genes and inversion in the chloroplast genome are reported to be useful in phylogenetic analyses to solve taxonomic problems at various taxonomic levels because they do not occur often and estimation of their homology and inversion event polarity is simple [22, 28–31]. With the importance of complete chloroplast genome in resolving phylogenetic relationship issues and the large number of genera and species in Acanthaceae only complete chloroplast genome of four genera has been so far reported (*Andrographis paniculata* (Burm.f.) Nees, NC_022451;* Ruellia breedlovei* T. F. Daniel, KP300014;* Strobilanthes cusia *(Nees) O. Kuntze, MG874806, and four species* Echinocactus *MF490441, MH045155, MH045156, and MH045157.)

In this study, we reported the characteristics of the complete chloroplast genome of* Justicia flava*, the first cp genome in the largest genus of Acanthaceae, and compared the genomes of four Acanthaceae species to understand the variation among the cp genomes, report the simple sequence repeats to provide the tools for genetic diversity and identification of the species, and lastly, resolve the status of Justiceae.

## 2. Materials and Methods

### 2.1. Plant Material and DNA Extraction

Plant material (vegetative and floral part) was collected through field survey of* J*.* flava* in Taif, Saudi Arabia, and identified based on the herbarium specimens and morphological features seen in relevant literatures, the voucher specimen was deposited in the herbarium of King Abdulaziz University, Jeddah, Saudi Arabia. Leaves were collected from the specimen for genomic DNA extraction. The genomic DNA was extracted using Plant Genomic DNA Kit according to manufacturer's protocol.

### 2.2. Library Construction, Sequencing, and Assembly

A total amount of 1.0*μ*g DNA was used as input material for the DNA sample preparations. Sequencing libraries were generated using NEBNext® DNA Library Prep Kit following manufacturer's recommendations and indices were added to each sample. The genomic DNA is randomly fragmented to a size of 350bp by shearing, then DNA fragments were end polished, A-tailed, and ligated with the NEBNext adapter for Illumina sequencing and further PCR enriched by P5 and indexed P7 oligos. The PCR products were purified (AMPure XP system) and resulting libraries were analyzed for size distribution by Agilent 2100 Bioanalyzer and quantified using real-time PCR. The qualified libraries are fed into Illumina sequencers after pooling according to its effective concentration and expected data volume. The raw reads were filtered to get the clean reads (5 Gb) using PRINSEQ lite v0.20.4 [32] and were subjected to de novo assembly using NOVOPlasty2.7.2 [33] with kmer (K-mer= 31) to assemble the complete chloroplast genome from the whole genome sequence*. ndhA*x1*-ndhA*x2 intergenic spacer from* Justicia flava* (KY632456) was used as seed and the plastome sequence of* Ruellia breedlovei* (KP300014.1) was used as the reference. Finally, one contig containing the complete chloroplast genome sequence was generated.

### 2.3. Gene Annotation

The programme DOGMA (Dual Organellar GenoMe Annotator, University of Texas at Austin, Austin, TX, USA) [34] was used to annotate the genes in the assembled chloroplast genome. The positions of start and stop codon were adjusted manually. trnAscan-SE server (http://lowelab.ucsc.edu/tRNAscan-SE/) [35] was used to verified the tRNA genes and finally, the plastome genome circular map was drawn using OGDRAW (Organellar Genome DRAW) [36]. The sequence of the chloroplast genome of* J. Flava* was deposited in the GenBank database with accession number (MK548577,).

### 2.4. Sequence Analysis

MEGA 6.0 was used to analyze the relative synonymous codon usage values (RSCU), base composition, and codon usage. Possible RNA editing sites present in the protein coding genes of* J. flava* cp genome were determined using PREP suite [37] with 0.8 as the cutoff value.

### 2.5. Repeat Analysis in* J. flava* Chloroplast Genome

Simple sequence repeats (SSRs) were identified in the* J*.* flava* chloroplast genome and genome of other three species of Acanthaceae using the online software MIcroSAtellite (MISA) [38] with the following parameters: eight, five, four, and three repeats units for mononucleotides, dinucleotides, trinucleotides, and tetra-, penta-, hexanucleotides SSR motifs, respectively. For analysis of long repeats (palindromic, forward, reverse, and complement) the program REPuter (https://bibiserv.cebitec.uni-bielefeld.de/reputer) [39] with default parameters was used to identify the size and location of the repeats in* J*.* flava* chloroplast genome and genome of other 3 species of Acanthaceae.

### 2.6. Genome Comparison

The program mVISTA [40] was used to compare the chloroplast genome of* J. flava* with the cp genomes of* E*.* longzhouensis, R*.* breedlovei*, and* S*.* cusia* using the annotation of* J. flava* as reference in the Shuffle-LAGAN mode [41]. The border region between the large single copy (LSC) and inverted repeat (IR) and small single copy SSC and inverted repeat (IR) junction were compared among the four species of Acanthaceae.

### 2.7. Characterization of Substitution Rate

DNAsp v5.10.01 [42] was used to analyze synonymous (dS) and nonsynonymous (dN) substitution rate and dN/dS ratio to detect the genes that are under selection pressure; the chloroplast genome of* J. flava* was compared with the cp genome of* E*.* longzhouensis, R*.* breedlovei,* and* S*.* cusia*. Individual protein coding genes were aligned separately in Genious V 8.1.3; the aligned sequences were then translated into protein sequence.

### 2.8. Phylogenetic Analysis

The complete chloroplast genome of three Acanthaceae and five species from the order Lamiales were downloaded from Genbank. The downloaded sequences were aligned with sequenced cp genome of* J*.* flava* using MAFFT v.7 [43]. The data were analyzed using Maximum Parsimony (PAUP version 4.0b10) [44] using heuristic searches with 1000 replicates of random taxon addition, tree bisection-reconnection branch swapping, MulTrees on, saving a maximum of 100 trees each replicate. Missing characters were treated as gaps. Support was assessed using 1000 replicates of nonparametric bootstrap analysis. Bayesian analysis was carried out using MrBayes version 3.2.6 [45]. jModelTest version 3.7 [46] was used to select the suitable model.

## 3. Results and Discussion

### 3.1. Characteristics of* J. flava* Chloroplast Genome

**Figure 1:**
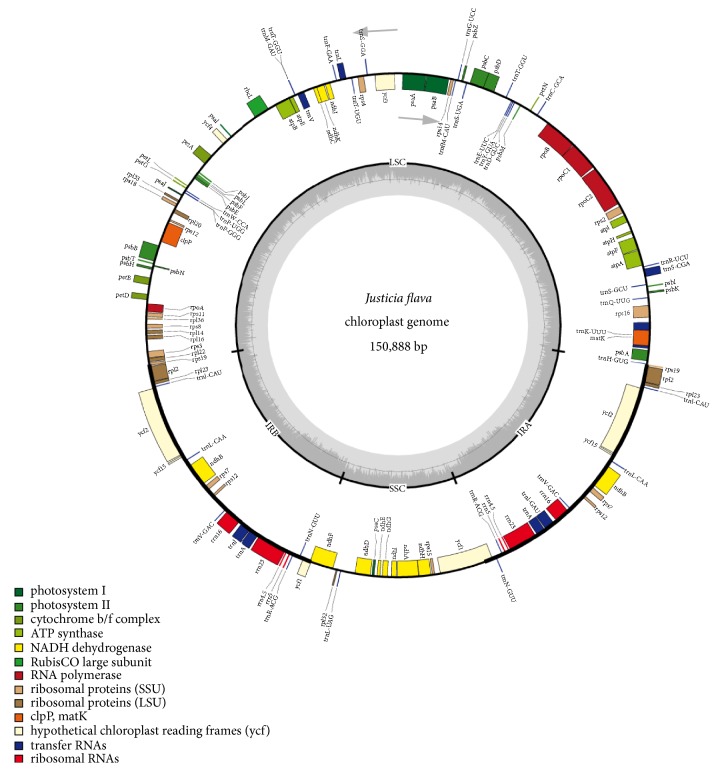
Gene map of the* J. flava* chloroplast genome. Genes outside the circles are transcribed in counterclockwise direction and those inside in clockwise direction. Known functional genes are indicated in the coloured bar. The GC and AT content are denoted by the dark grey and light grey colour in the inner circle, respectively. LSC indicates large single copy; SSC, indicates small single copy, and IR indicates inverted repeat.

The complete chloroplast genome of* J*.* flava* is a circular molecule and has quadripartite structure; the genome was found to have 150, 888bp in length. The genome is divided into four regions, namely, large single copy (LSC), small single copy (SSC), and two inverted repeats (IRa and IRb). The coding region is 98, 671bp in length and constitutes 65.39% of the genome; the rest of 52, 217 bp is the intergenic spacer region including intron (34.60%). The LSC and SSC regions possessed 82, 995bp and 16, 893bp, respectively, the inverted repeats IRa and IRb have 25, 500bp and are separated by SSC region (Figure 1 and Table 1). The organization and structure of the* J*.* flava* cp genome are similar to other sequenced Acanthaceae cp genomes [47, 48]. The percentage of occurrence of AT and GC content in the genome showed that the LSC, SSC, IRA, and IRB regions possessed 63.8% and 36.2%, 67.8% and 32.2%, 56.7% and 43.4%, and 56.7% and 3.4, respectively. The whole chloroplast genome has AT content of 61.8% and GC content of 38.2%; this is similar to cp genome of* Strobilanthes cusia* [49]. The GC content in the inverted repeat is found to be higher than the single copy regions both the LSC and SSC.

The result of the genes annotation in the chloroplast genome of* J. flava* revealed a total of 132 genes among which 113 are unique; the remaining 19 are duplicated in the inverted region. The genome harbored 80 protein coding genes, 30 tRNA genes, and 4 rRNA genes (Figure 1 and Table 2). The numbers and orientation of the genes in the cp genome are the same as other cp genomes of Acanthaceae [47, 48]. The inverted repeat region contained eight protein coding genes, seven tRNA, and four rRNA while in the single copy region, the LSC contained 61 protein coding genes and 22 tRNA genes; the rest of 12 protein coding genes and 1 tRNA are located within the SSC region. Almost all the protein coding genes start with the ATG codon that code for methionine whereas some of the genes start with codon like ATC, GTG and ACG; this is common in most flowering plant (angiosperms) chloroplast genome [49–51].

The* J. flava* chloroplast genome is found to contain intron in some of the protein coding and tRNA genes, like other chloroplast genomes of angiosperms [49, 50]. There are 14 genes that contain intron out of the 113 different genes (Table 3); among the 14 genes 8 are protein coding genes while the remaining six are tRNA genes (Table 3). Four genes that have the intron, namely,* rpl2*,* ndhB*,* trnI-GAU, *and* trnA-UGC *are located in the IR region while the remaining 12 are located in the LSC region. Only two genes* ycf3* and* clpP* have two introns, the other 12 have only one intron, and this is also seen in* S. cusia* [49]. The tRNA,* trnK-UUU *has the longest intron of 2460 bp (Table 3); this is as a result of position of the* matK* gene in the intron.

**Figure 2:**
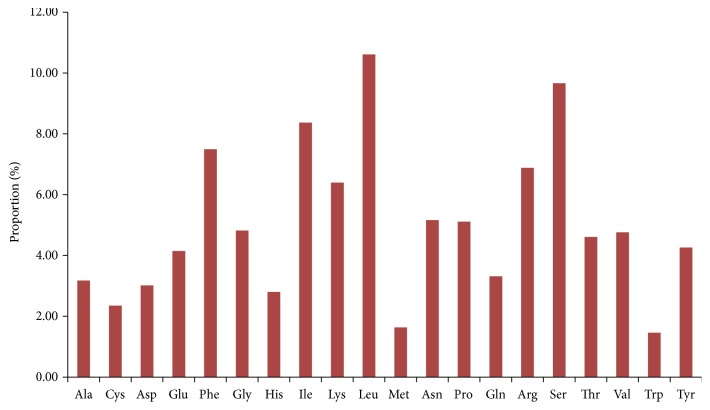
Amino acids frequencies in* J. flava* chloroplast genome protein coding sequences.

The codon usage bias in the plastome was computed using the protein coding genes and tRNA genes nucleotide sequences 89, 377bp. The relative synonymous codon usage of each codon in the genome is presented in (Table 4); the result revealed that all the genes are encoded by 29, 790 codons. Codons, coding for the amino acids Leucine, are the most frequent codons 3,329 (11.7%) (Figure 2), similar to that of* Ailanthus altissima* [52], whereas codons coding for Trp are the least 570 (1.91%) in the genome. G- and C-ending are found to be more frequent than their counterpart A and T; this is not the case in other plastomes sequences [53–55]. The result of the analysis (Table 4) showed that codon usage bias is low in the chloroplast genome of* J*.* flava*. The RSCU values of 29 codons were >1 and all of them have A/T ending while for 30 codons were <1 and are all of G/C ending. Only two amino acids Tryptophan and Methionine have RSCU value of 1; therefore they are the only amino acids with no codon bias.

The RNA editing site in the* J. flava *chloroplast genomes was predicted using the program PREP suite; all the analysis was done using the first codon position of the first nucleotide. The result (Table 5) shows that the majority of the conversion in the codon positions is from the amino acid Serine to Leucine (Table 5). In all, the programme revealed 61 editing sites in the genome; the editing sites are distributed among 18 protein coding genes. As reported in previous researches [56–58] the* ndhB* gene has the highest number of editing sites (9 sites) followed by* rpoB* (7 site) and* ndhG*,* atpF*,* rpl2*,* rpl20*, and* rps2* have the least 1 site each. Among all the conversion in the RNA editing site, one site changed the amino acid from apolar group to polar group (Proline to Serine). The following genes do not have RNA predicting site in their first codon of the first nucleotides* atpB, ccsA, clpP, ndhC, ndhE psaB, petD, petG, rpoA, and petL,* among others.

### 3.2. Repeat Analysis

#### 3.2.1. Long Repeats

REPuter programme was used to identify the repeat sequence in the chloroplast genome of* J. flava* using default settings; the result obtained showed that all the four types of repeats (palindromic, forward, reverse, and complement) were present in the genome (Table 6). The analysis showed 16 palindromic repeats, 23 forward repeats, 9 reverse repeats, and only one complement repeat (Table 6). Majority of the repeats size is between 20 and 29bp (46.93%), followed by 10-19bp (40.81%), whereas 30-39bp and 40-49bp are the least with 8.16% and 4.08%, respectively. In total, there are 49 repeats in the chloroplast genome of* J*.* flava*. In the first location the intergenic spacer harbored 65.30% of the repeats; this has also been reported in cp genome of* Fagopyrum dibotrys* [59]. tRNA contained 8 repeats (16.32%); the remaining 9 repeats (18.36%) are located in the protein coding genes specifically* atpB*,* psaB*,* ndhC*,* ycf1,* and* ycf2*. Within the protein coding genes* ycf2* contained 1 reverse and 2 palindromic and forward repeats.

**Figure 3:**
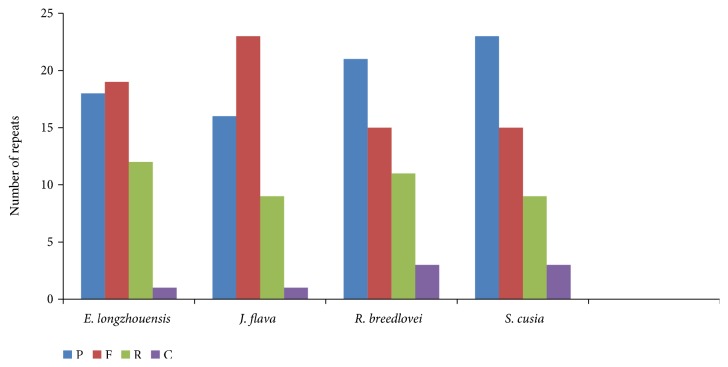
Number of different repeats in four chloroplast genome of Acanthaceae. P= palindromic, F = forward, R=reverse, and C= complement.

We compared the frequency of repeats among four Acanthaceae cp genomes and found that all the types of repeats (palindromic, forward, reverse, and complement) are present in all the genomes (Figure 3).* S*.* cusia *has the highest frequency of palindromic repeats (23) while* J. flava* has the lowest with (16).* R. breedlovei* and* S. cusia* have the same number of forward repeats 15 each, and number of reverse repeats is the same in the genome of* J. flava* and* S. cusia *(Figure 3). Complement repeats are found to be the less type of repeat in the genome with* E. longzhouensis* and* J. flava* having 1 and the other two species having 3 each.

#### 3.2.2. Simple Sequence Repeats (SSRs)

**Figure 4:**
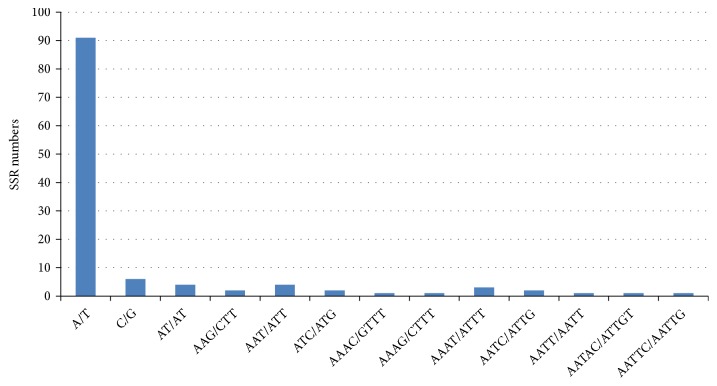
Frequency of different SSR motifs in different repeat types in* J. flava* chloroplast genome.

**Figure 5:**
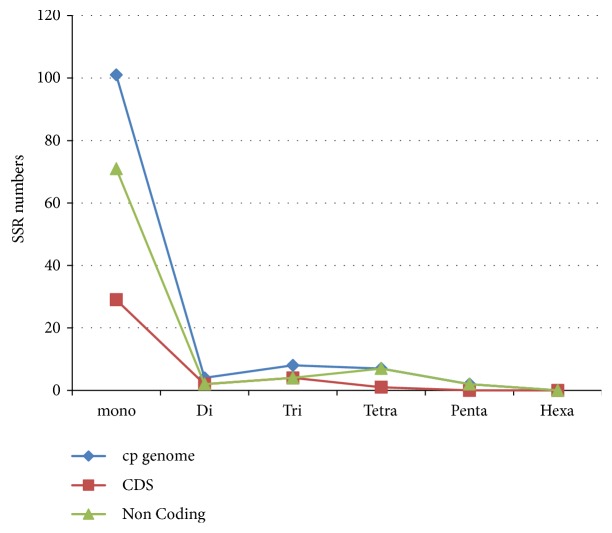
Number of SSR types in complete genome, protein coding regions, and noncoding genes.

There are short repeats of nucleotide series (1-6 bp) that are dispensed all through genome called microsatellites (SSRs). This short repeat in plastid genome is passed from a single parent. As a result, they are used as molecular indicators in developmental studies such as genetic heterogeneity and also contribute in recognition of species [60–62]. The sums of 98 microsatellites were found in plastid genome of* J. flava* in this study (Table 7). Majority of SSRs in the cp genome are mononucleotide (83.67%) of which most are poly T and A (Figure 4). Poly T (polythymine) constituted 58.53% whereas poly A (polyadenine) 40.24%; this is consistent in previous studies [63, 64]. Only a single poly C (polycytosine) 1.21% is present in the genome whereas 2 poly G (polyguanine) is found in the genome. Among the dinucleotide only AT/AT is found in the genome. Reflecting series complementary, three trinucleotide AAG/CTT, ATC/ATG, and AAT/ATT, five tetra AAAC/GTTT, AAAG/CTTT, AAAT/ATTT, AATC/ATTG, and AATT/AATT, and two penta AATAC/ATTGT and AATTC/AATTG were discovered in the genome while no hexanucleotide repeat is present (Figure 4). The intergenic spacer region harbored most of the microsatellite (62.24%) than the coding region (33.67%) (Figure 5). Most but not all the repeats (70.40%) were detected in the LSC region and the SSC region incorporates the least number of repeats in the genome.

**Figure 6:**
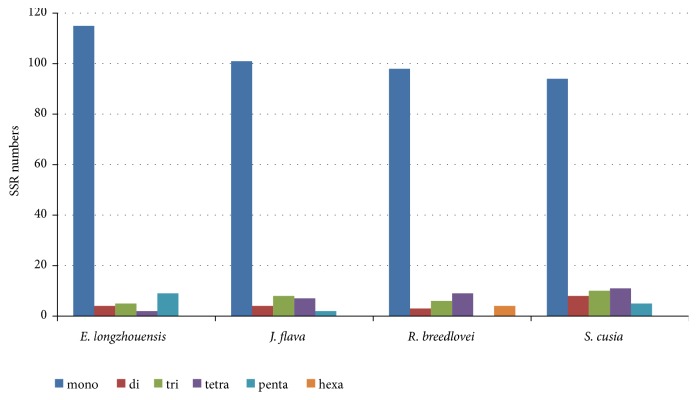
Number of different SSR types in the four chloroplast genome of Acanthaceae.

The frequency of SSR among the cp genome of the four species was also compared (Figure 6); the comparison showed that mononucleotide occurs more frequently across all the genomes.* E. longzhouensis* had the highest number of mononucleotides and pentanucleotides with 115 and 9, respectively, but had the lowest number of tetranucleotides with 2. Pentanucleotide is not present in cp genome of* R. breedlovei* while possessing hexanucleotide which is not present in the remaining 3 species.

### 3.3. Comparative Analysis of* J. flava* Chloroplast to Other Acanthaceae Genomes

**Figure 7:**
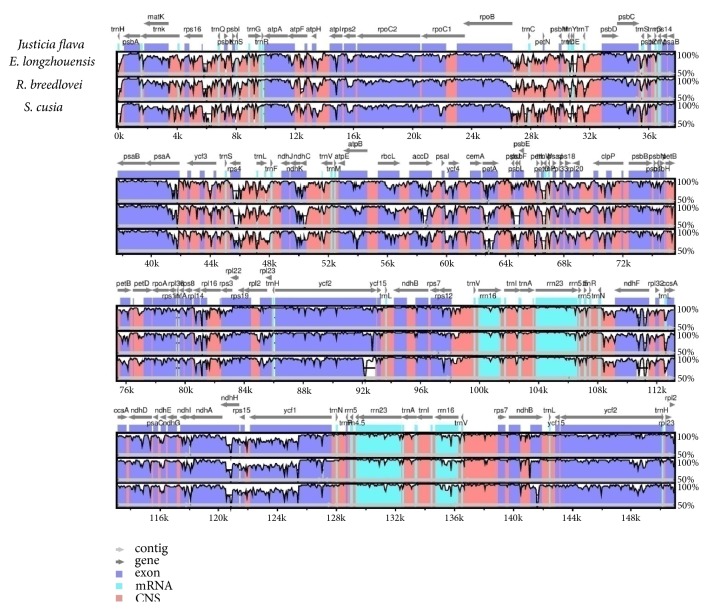
Sequence alignment of four chloroplast genomes in the Acanthaceae family performed with mVISTA using annotation of* J. flava *as reference. The top arrow shows transcription direction, blue colour indicates protein coding, pink colour shows conserved noncoding sequence CNS, and light green indicates tRNAs and rRNAs. The x-axis represents the coordinates in the cp genome while y-axis represents percentage identity within 50-100%.

The complete chloroplast genome of* J*.* flava* was compared with three chloroplast genomes of Acanthaceae available in the Genbank, namely,* R. breedlovei*,* S. cusia,* and* E*.* longzhouensis*. To examine the degree of DNA sequence divergence among the species of Acanthaceae chloroplast genome, the programme mVISTA was used to align the sequences using annotation of Justicia flava as reference. The result of the alignment showed that the genomes highly conserved with some degree of divergence. The inverted repeat regions are more conserved than the single copy regions, the large single copy and small single copy; on the other hand the protein coding genes were found to be more conserved than the noncoding region, particularly the intergeneric spacer. The nonprotein coding regions that showed high rate of divergence across the genome are* trnH-GUG*-*psbA*,* rps16*-*trnQ*,* trnC*-*petN*,* accD*-*psaI*,* clpP* intron,* trnL*-*trnF*,* rps15*-*ycf1*,* rps12*-*trnV*, and* trnL*-*trnA* among others (Figure 7). For the protein coding genes the following genes showed a little sequence variation among the genomes* atpE, atpF, rbcL, petA, psbL, petB, and ycf2*.

**Figure 8:**
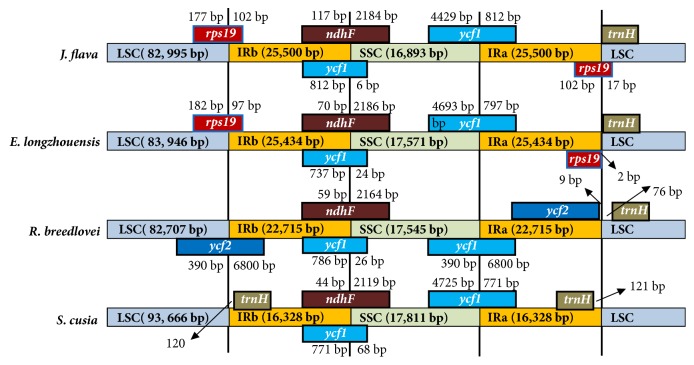
Comparison of the borders of the IR, SSC, and LSC regions among four chloroplast genome of Acanthaceae.

The chloroplast genome of angiosperms is reported to be conserved in terms of structure and size [65]; despite the conserved nature there is slightly variation in size and the boundaries of inverted repeats and single copy regions due to evolutionary events such as expansion and contraction in the genome [66, 67]. The comparisons between IR-LCS and IR-SSC boundaries in the four cp genome of Acanthaceae (*Justicia flava, Echinocactus longzhouensis, Ruellia breedlovei,* and* Strobilanthes cusia*) are shown in (Figure 8). The result showed that there is slightly variation among the compared cp genomes (Figure 8); four genes, namely,* rps19*,* ndhF*,* ycf1,* and* trnH,* were located in the junction of inverted repeats and single copy region of* J. flava* and* E. longzhouensis *genome with slightly variation in number of base pairs in the borders (Figure 8).Two genes,* ndhF* and* ycf1,* are found in the IRb/SSC border among all the four genomes. The IRb/LCS border of* R*.* breedlovei* is unique by having* ycf2* gene with 390 bp in LSC and 6800 bp in IRb whereas Strobilanthes cusia genome also has unique structural variation by having trnH in IRa and IRb. The* ndhF* was found to have 70 bp, 59 bp, and 44 bp in the IRb region in* J. flava, E. longzhouensis, R. breedlovei, and S. cusia,* respectively, whereas the* trnH* of* J. flava* and* E. longzhouensis* starts at exactly IRa/LSC border while the tRNA is 76 bp away the border in* R. breedlovei* genome.

### 3.4. Divergence of Protein Coding Gene Sequence

**Figure 9:**
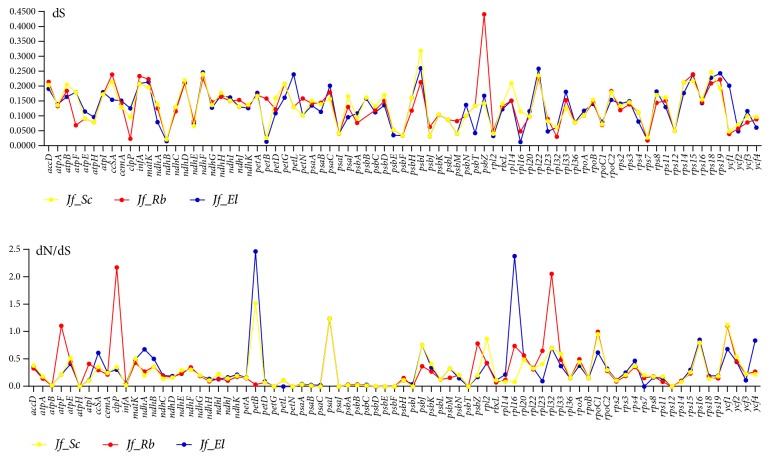
The synonymous (dS) and dN/dS ration values of 78 protein coding genes from four Acanthaceae cp genomes (*Jf*:* J*.* flava; Rb*:* R. breedlovei*;* El*:* E. longzhouensis*).

The rates of synonymous (dS) and nonsynonymous (dN) substitution and dN/dS ratio were calculated to detect the selective pressure among the 78 protein coding genes in the cp genome of four Acanthaceae species. The results showed that the dN/dS ration is less than 1 in most of the paired genes except* petB*,* psaI,* and* ycf1* of* J. flava* vs.* S. cusia* having 1.52, 1.24, and 1.12, respectively (Figure 9). Two genes are also found to be greater than 1 in* J. flava* vs.* R. breedlovei atpF*,* clpP*,* psaI,* and* rpl32*.* petB*,* rpl16,* and* psaI *genes of* J. flava* vs.* E. longzhouensis* are greater than 1 as well. This indicates that most of the genes were under negative selection; only few undergo positive selection. The synonymous (dS) values in all the genes range from 0.02 to 0.44 (Figure 9). The genes,* atpH, petG, petN, psaC, psaJ, psbE, psbF, psbI, psbT,* and* rps12, *showed no nonsynonymous change occurs in the cp genome of the four species of Acanthaceae.

### 3.5. Phylogenetic Analysis

**Figure 10:**
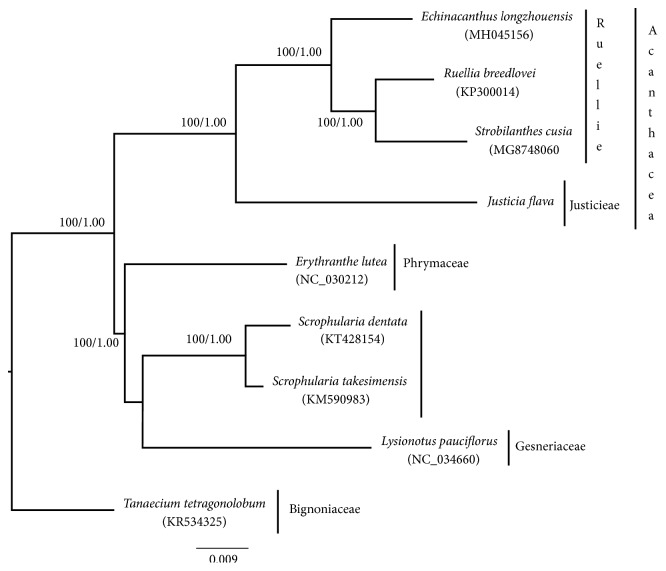
Phylogenetic tree reconstruction of 9 taxa based on the complete chloroplast genome using Bayesian Inference (BI) and Maximum Parsimony (MP) methods showing relationship within the four species of Acanthaceae. The numbers in the branch nodes represent bootstrap percentage (BP)/posterior probability (PP).

The phylogenetic relationship within the four species of Acanthaceae was reconstructed using the complete chloroplast genome. The tree from the Bayesian Inference and Maximum Parsimony is congruent with strong support in all the nodes PP, 1.00, and MP, 100. All the species of Acanthaceae sampled clustered in one clade with strong support (Figure 10), as reported by [68]. The result showed that the tribe Justicieae is sister to Ruellieae; this relationship has also been reported by [69] and they should be regarded as independent tribe not as Justiceae under the tribe Ruellieae as proposed by [11]

## Figures and Tables

**Table 1:** Base composition in the *J. flava *chloroplast genome.

Region		T(U) (%)	C (%)	A (%)	G (%)	Total (bp)
cp genome		31	19	31	19	150888
LSC		32	19	31	18	82995
SSC		34	17	34	15	16893
IRA		28	23	28	21	25500
IRB		28	21	28	23	25500
	1st Position	31	20	30	19	50296
	2nd Position	31	19	31	19	50296
	3rd Position	31	20	30	19	50296

**Table 2:** Genes present in the chloroplast genome of *J. flava*.

Category	Group of genes	Name of genes
RNA genes	ribosomal RNA genes (rRNA)	*rrn5, rrn4.5, rrn16, rrn23*

	Transfer RNA genes (tRNA)	*trnH-GUG, trnK-UUU* ^+^, *trnQ-UUG, trnS-GCU, trnS-CGA*^+^, *trnR-UCU,trnC-GCA, trnD-GUC, trnY-GUA, trnE-UUC, trnT-GGU, trnS-UGA, trnfM-CAU, trnG-GCC, trnS-GGA, trnL-UAA*^*+*^*, trnT-UGU, trnF-GAA, trnV-UAC*^+^, *trnM-CAU*, *trnW-CCA, trnP-UGG, trnI-CAU*^a^*, trnL-CAA*^a^,* trnV-GAC*^a^, *trnI-GAU*^+,a^,* trnA-UGC*^+,a^, *trnR-ACG*^a^, *trnN-GUU*^a^, *trnL-UAG*,

Ribosomal proteins	Small subunit of ribosome	*rps2, rps3, rps4, rps*7^a^, *rps8, rps11, rps*12^a^, *rps14*, *rps15*,* rps,16*^+^, *rps18,rps19*

Transcription	Large subunit of ribosome	*rpl*2^+,a^, *rpl14, rpl16, rpl20, rpl22, rpl*23^a^, *rpl32, rpl33, rpl36*

	DNA dependent RNA polymerase	*rpoA, rpoB, rpoC1* ^+^, *rpoC2*

Protein genes	Photosystem I	*psaA, psaB, psaC, psaI,psaJ,ycf3* ^++^

	Photosystem II	*psbA, psbB, psbC, psbD, psbE, psbF, psbH, psbI, psbJ, psbK, psbL, psbM, psbN, psbT, psbZ*

	Subunit of cytochrome	*petA, petB, petD, petG, petL, petN*

	Subunit of synthase	*atpA, atpB, atpE, atpF* ^+^, *atpH, atpI*

	Large subunit of rubisco	*rbcL*

	NADH dehydrogenase	*ndhA* ^+^,*ndhB*^+a^, *ndhC, ndhD, ndhE, ndhF*, *ndhG, ndhH, ndhI, ndhJ, ndhK*

	ATP dependent protease subunit P	*clpP* ^++^

	Chloroplast envelope membrane protein	*cemA*

Other genes	Maturase	*matK*

	Subunit acetyl-coA carboxylase	*accD*

	C-type cytochrome synthesis	*ccsA*

	Hypothetical proteins	*ycf*2^a^, *ycf4*, *ycf*15^a^

	Component of TIC complex	*ycf*1^a^

^+^Gene with one intron. ^++^Gene with two intron. ^a^ Gene with copies.

**Table 3:** Genes with intron the *J. flava* chloroplast genome and length of introns and exons.

Gene	Location	Exon I (bp)	Intron I (bp)	Exon II (bp)	Intron II (bp)	Exon III (bp)
*atpF*	LSC	143	659	467		
*rpoC1*	LSC	443	769	1628		
*ycf3*	LSC	128	682	227	721	152
*clpP*	LSC	68	763	293	635	227
*rpl2*	IR	392	664	434		
*ndhB*	IR	776	680	755		
*ndhA*	SSC	551	954	539		
*trnK-UU*	LSC	36	2460	37		
*trnS-CGA*	LSC	31	667	59		
*trnL-UAA*	LSC	36	501	49		
*trnV-UAC*	LSC	37	593	36		
*trnI-GAU*	IR	41	938	34		
*trnA-UGC*	IR	37	818	34		

**Table 4:** Codon-anticodon recognition patterns and codon usage of the *J. flava* chloroplast genome.

Codon	Amino Acid	RSCU	tRNA	Codon	Amino Acid	RSCU	tRNA
UUU	Phe	1.18	*trnF-GAA*	UAU	Tyr	1.38	*trnY-GUA*
UUC	Phe	0.82		UAC	Tyr	0.62	
UUA	Leu	1.31	*trnL-UAA*	UAA	Stop	1.01	
UUG	Leu	1.31	*trnL-CAA*	UAG	Stop	1.03	
CUU	Leu	1.23	*trnL-UAG*	CAU	His	1.26	*trnH-GUG*
CUC	Leu	0.65		CAC	His	0.74	
CUA	Leu	0.92		CAA	Gln	1.37	*trnQ-UUG*
CUG	Leu	0.59		CAG	Gln	0.63	
AUU	Ile	1.22	*trnI-GAU*	AAU	Asn	1.34	*trnG-GUU*
AUC	Ile	0.82		AAC	Asn	0.66	
AUA	Ile	0.95	*trnI-CAU*	AAA	Lys	1.29	*trnK-UUU*
AUG	Met	1	*trnM-CAU*	AAG	Lys	0.71	
GUU	Val	1.45	*trnV-GAC*	GAU	Asp	1.44	*trnD-GUC*
GUC	Val	0.65		GAC	Asp	0.56	
GUG	Val	0.74		GAA	Glu	1.38	*trnE-UUC*
GUA	Val	1.16	*trnV-UAC*	GAG	Glu	0.62	
UCU	Ser	1.46	*trnS-GGA*	UGU	Cys	1.14	*trnC-GCA*
UCC	Ser	0.95		UGC	Cys	0.86	
UCG	Ser	0.76		UGA	Stop	0.96	
UCA	Ser	1.33	*trnS-UGA*	UGG	Trp	1	*trnW-CCA*
CCU	Pro	1.21	*trnP-UGG*	CGU	Arg	0.84	*trnR-ACG*
CCC	Pro	0.87		CGC	Arg	0.35	*trnR-UCU*
CCA	Pro	1.13		CGA	Arg	1.16	
CCG	Pro	0.79		CGG	Arg	0.77	
ACU	Thr	1.22		AGA	Arg	1.8	
ACC	Thr	0.87		AGG	Arg	1.07	
ACG	Thr	0.72	*trnT-GGU*	AGU	Ser	0.92	*trnS-GCU*
ACA	Thr	1.2	*trnT-UGU*	AGC	Ser	0.58	
GCU	Ala	1.33	*trnA-UGC*	GGU	Gly	1.18	*trnG-GCC*
GCC	Ala	0.76		GGC	Gly	0.54	
GCA	Ala	1.18		GGA	Gly	1.28	
GCG	Ala	0.72		GGG	Gly	0.99	*trnG-UCC*

**Table 5:** Predicted RNA editing site in the *J. flava* chloroplast genome.

gene	Nucleotide Position	Amino Acid Position	Codon Conversion	Amino Acid Conversion	Score
*accD*	800	267	TCG => TTG	S => L	0.8
	844	282	CCC => TCC	P => S	0.8
*atpA*	776	259	ACC => ATC	T => I	1
	914	305	TCA => TTA	S => L	1
	1270	424	CCC => TCC	P => S	1
*atpF*	92	31	CCA => CTA	P => L	0.86
*atpI*	404	135	GCT => GTT	A => V	1
	620	207	TCA => TTA	S => L	1
*matK*	640	214	CAT => TAT	H => Y	1
	1249	417	CAT => TAT	H => Y	1
*ndhA*	326	109	ACT => ATT	T => I	1
	566	189	TCA => TTA	S => L	1
	922	308	CTT => TTT	L => F	1
*ndhB*	149	50	TCA => TTA	S => L	1
	467	156	CCA => CTA	P => L	1
	586	196	CAT => TAT	H => Y	1
	737	246	CCA => CTA	P => L	1
	746	249	TCT => TTT	S => F	1
	830	277	TCA => TTA	S => L	1
	836	279	TCA => TTA	S => L	1
	1292	431	TCC => TTC	S => F	1
	1481	494	CCA => CTA	P => L	1
*ndhD*	2	1	ACG => ATG	T => M	1
	32	11	GCA => GTA	A => V	1
	878	293	TCA => TTA	S => L	1
	1445	482	GCT => GTT	A => V	1
*ndhF*	124	42	CTT => TTT	L => F	1
	671	224	CCA => CTA	P => L	1
	713	238	GCT => GTT	A => V	0.8
	1505	502	TCT => TTT	S => F	1
	1667	556	CCC => CTC	P => L	1
	2173	725	CTC => TTC	L => F	1
*ndhG*	314	105	ACA => ATA	T => I	0.8
*petB*	617	206	CCA=> CTA	P => L	1
*psaI*	22	8	CCT => TCT	P => S	1
	28	10	CTT => TTT	L => F	0.86
*rpl2*	596	199	GCG => GTG	A => V	0.86
*rpl20*	308	103	TCA => TTA	S => L	0.86
*rpoB*	338	113	TCT => TTT	S => F	1
	473	158	TCA => TTA	S => L	0.86
	551	184	TCA => TTA	S => L	1
	566	189	TCG => TTG	S => L	1
	593	198	GCT => GTT	A => V	0.86
	2000	667	TCT => TTT	S => F	1
	2426	809	TCA => TTA	S => L	0.86
*rpoC2*	2290	764	CGG => TGG	R => W	1
	3202	1068	CTT => TTT	L => F	0.86
	3719	1240	TCA => TTA	S => L	0.86
*rps2*	248	83	TCA => TTA	S => L	1
	266	89	ACA => ATA	T => I	0.86
*rps14*	80	27	TCA => TTA	S => L	1

**Table 6:** Repeat sequences present in the *J. flava* chloroplast genome.

S/N	Repeat Size	Repeat Position 1	Repeat Type	Repeat Location 1	Repeat Position 2	Repeat Location 2	E-Value
1	41	97201	F	IGS	117423	IGS	1.32E-15
2	41	117423	P	IGS	136591	IGS	1.32E-15
3	39	42922	F	*ycf3* Intron	97203	IGS	2.12E-14
4	39	42922	F	*ycf3* Intron	117425	IGS	2.12E-14
5	39	42922	P	*ycf3* Intron	136591	IGS	2.12E-14
6	30	7623	P	IGS-*trnS-GCU*	44420	IGS-*trnSGCA*	5.55E-09
7	26	86664	P	*ycf2*	86664	*ycf2*	1.42E-06
8	26	86664	F	*ycf2*	147143	*ycf2*	1.42E-06
9	26	120785	F	IGS	120808	IGS	1.42E-06
10	26	147143	P	*ycf2*	147143	*ycf2*	1.42E-06
11	25	118049	F	ndhA Intron	118074	*ndhA* Intron	5.69E-06
12	24	58638	F	IGS	58661	IGS	2.27E-05
13	23	31057	R	IGS	31057	IGS	9.10E-05
14	22	9120	F	*trnG-GCC*	35746	*trnG-UCC*	3.64E-04
15	21	7629	F	*trnS-GCU*-IGS	34812	*trnS-UGA*	1.46E-03
16	21	11762	R	IGS	11762	*IGS*	1.46E-03
17	21	34812	P	*trnS-UGA*	44423	trnS-GGA	1.46E-03
18	21	45956	R	*trnT-UGU*	45956	trnT-UGU	1.46E-03
19	21	106777	F	IGS	123747	*ycf1*	1.46E-03
20	21	123747	P	*ycf1*	127035	IGS	1.46E-03
21	20	29704	R	IGS	29704	IGS	5.82E-03
22	20	30622	P	IGS	30622	IGS	5.82E-03
23	20	49490	R	*ndhC*	49490	*ndhC*	5.82E-03
24	20	51290	P	*trnV-UAC*	101924	*trnA-UGC*	5.82E-03
25	20	51290	F	*trnV-UAC*	131889	*trnA-UGC*	5.82E-03
26	20	92988	F	IGS	93005	IGS	5.82E-03
27	20	92988	P	IGS	140808	IGS	5.82E-03
28	20	93005	P	IGS	140825	IGS	5.82E-03
29	20	140808	F	IGS	140825	IGS	5.82E-03
30	19	11760	P	IGS	80056	IGS	2.33E-02
31	19	43979	F	IGS	43997	IGS	2.33E-02
32	19	58511	R	IGS	58511	IGS	2.33E-02
33	19	108471	R	*ycf2*	108471	*ycf2*	2.33E-02
34	19	120905	R	IGS	120905	IGS	2.33E-02
35	19	123419	F	*ycf1*	123443	*ycf1*	2.33E-02
36	18	235	P	IGS	269	IGS	9.32E-02
37	18	5052	F	IGS	5069	IGS	9.32E-02
38	18	7694	F	*trnS-GCU*	34882	*trnS-UGA*IGS	9.32E-02
39	18	7758	P	IGS	67000	IGS	9.32E-02
40	18	11761	F	*atpF* Intron	54061	IGS	9.32E-02
41	18	21857	F	*rpoC1* Intron	27884	IGS	9.32E-02
42	18	30254	P	IGS	30254	IGS	9.32E-02
43	18	30895	R	IGS	30895	IGS	9.32E-02
44	18	37376	F	*psaB*	39591	*psaA*	9.32E-02
45	18	37925	F	*psaB*	40149	*psaA*	9.32E-02
46	18	46008	P	IGS	46008	IGS	9.32E-02
47	18	50038	C	IGS	80055	IGS	9.32E-02
48	18	53019	F	*atpB*	125148	*ycf1*	9.32E-02
49	18	54060	F	IGS	106779	IGS	9.32E-02

**Table 7:** Simple sequence repeats in the chloroplast genome of *J*. *flava.*

Repeat	Length (bp)	Number	Start position
A	8	17	1, 941; 4,077; 7, 866; 11, 688; 13, 037; 17, 946; 41, 684; 43, 752; 51, 259; 54, 200; 68, 846; 66, 730; 95, 631; 110, 063; 111, 010; 113, 860; 118, 261; 150, 742; 150, 809
	11	3	7, 592; 14, 787; 15, 599
	10	3	14, 665; 21, 867; 28, 269;
	9	8	27, 894; 43, 551; 45, 670; 63, 290; 88, 481; 114, 354; 132, 510; 150, 784
	14	1	80, 057;
	13	1	127, 037;

C	12		4, 487

G	8	2	57, 723; 74, 644

T	8	25	7, 383; 25, 533; 32, 688; 59, 519; 60, 372; 65, 495; 66, 271; 66, 377; 68, 096; 68, 556; 74, 497; 81, 050; 82, 410; 82, 710; 83, 018; 83, 085; 109, 106; 109, 136; 112, 173; 112, 563; 113, 364; 123, 499; 123, 541; 125, 326; 138, 196
	10	4	9, 482; 30, 515; 53, 621; 123, 624;
	15	1	11, 766;
	9	15	12, 488; 15, 906; 17, 805; 70, 662; 74, 580; 75, 746; 81, 096; 83,050; 101, 316; 109, 159; 121, 849; 123, 004; 123, 606; 123, 638; 145, 345
	11	3	30, 883; 31, 611; 123, 292;
	12	3	35, 533; 77, 108; 124, 143
	14	2	50, 040; 54, 066;
	13	2	106, 785; 123, 755

AT	6	1	7, 215
	5	1	20, 206

TA	5	2	19, 175; 30, 628

TTC	4	1	34, 429

TAT	4	1	84991

TGA	4	1	90, 256

TCT	5	1	124, 548

ATC	4	1	143, 566

ATA	4	1	148, 832

TAA	4	1	62, 980

TTTC	3	1	5, 222

ATTG	3	1	5, 410

ATAA	3	1	58, 759

TAAA	4	1	66, 121

AAAC	3	1	67, 128

AATA	3	1	112, 973

AATC	3	1	118, 083

AATT	3	1	122, 503

CAATA	3	1	30, 293

## Data Availability

(1) The complete chloroplast genome sequence can be found in Genbank with accession no. MK548577 after publishing the article. (2) The data used to support the findings of this study are available from the corresponding author upon request.
