# Corrigendum to “Complete Chloroplast Genome Sequence of *Justicia flava*: Genome Comparative Analysis and Phylogenetic Relationships among Acanthaceae”

**DOI:** 10.1155/2020/2049759

**Published:** 2020-07-16

**Authors:** Samaila S. Yaradua, Dhafer A. Alzahrani, Enas J. Albokhary, Abidina Abba, Abubakar Bello

**Affiliations:** ^1^Department of Biology, King Abdulaziz University, Jeddah, Saudi Arabia; ^2^Centre for Biodiversity and Conservation, Department of Biology, Umaru Musa Yaradua University, Katsina, Nigeria

In the article titled “Complete Chloroplast Genome Sequence of *Justicia flava*: Genome Comparative Analysis and Phylogenetic Relationships among Acanthaceae”, the funding was omitted in error. The statement is shown below:

“This project was funded by the Deanship of Scientific Research (DSR), King Abdulaziz University, Jeddah, under grant no. D-119-130-1441. The authors, therefore, gratefully acknowledge DSR technical and financial support.”
